# The use of inhaled corticosteroid in preschool wheezers: what's the point today?

**DOI:** 10.1186/1824-7288-35-43

**Published:** 2009-12-24

**Authors:** Laura Tenero, Giulia Paiola, Alessandra Coghi, Giorgio L Piacentini

**Affiliations:** 1 Clinica Pediatrica - Università di Verona, Verona, Italy

## Abstract

Among the preschool children who wheeze two different groups can be identify: children who have a viral infection and those who respond to multiple triggers, such as exercise or allergens.

To distinguish between these different phenotypes of wheezing, and consequently choose therapy represents a major challenge for pediatricians.

Transient wheezers conditions do not improve with maintenance treatment with ICS. On the other hand they are definitely useful in children with wheeze/asthma.

Increasing evidence is in favor of the potential role of leukotriene receptor antagonists in preschool children with recurrent wheezing.

Oral steroid has been demonstrated not to be indicated to control acute wheezing, unless severe disease is expected in non-atopic children.

The early phenotyping of preschool wheezers, upon which the appropriate treatment should be based, represents a challenging issue in the paediatric practice.

## Phenotyping of the wheezing

Wheezing and cough are frequently observed in preschool children, in many cases related to a respiratory tract infection.

These situations represent a major challenge in the practice of paediatricians since, at present, it is very hard to distinguish between the different phenotypes which underlie an apparently similar clinical presentation.

In fact, among the preschool children who wheeze, at least two different groups can be identified, i.e. children who wheeze on the basis of a viral trigger and those who respond to multiple triggers, the latter being somehow referable to the asthmatic wheezing [1].

The temporal pattern for viral wheezing is commonly reported as episodic, with periods free of symptoms between episodes, and is reported to be concomitant with a viral infection of the airways.

In contrast, children with multiple trigger wheezing, though being highly responsive to viral infections which represent the most common trigger for their symptoms, also wheeze when exposed to a variety of other triggers, such as exercise or allergens [1].

Cohort studies have suggested that some clinical characteristics can help in distinguishing among the different phenotypes of preschool wheezers [2], but the application of such parameters cannot be considered conclusive for the clinical purposes.

More recently, studies based upon a latent class analysis have demonstrated that considering clusters of symptoms may contribute to the identification of different phenotypes among children with respiratory disorders [3].

## The clinical approach to preschool wheezers

From a practical point of view, the difficulty in allocating an individual patient within a specific phenotype represent a major task for paediatricians not only in terms of diagnostic procedures but consequently also, and even more importantly, in terms of treatment strategy.

In particular, in the last few months, a body of discussion has been raised around the issue of the use of corticosteroids, either systemic or inhaled in the treatment of acute wheezing in preschool children.

The use of inhaled or oral corticosteroids in preschool wheezers is widely diffuse in the paediatric practice, mainly based upon the recommendation of guidelines for elder asthmatic children.

Some recent publications has looked for a deeper insight into the challenging issue of the use of either systemic or inhaled corticosteroids in younger children with wheezing.

In particular, Panickard et al have studied the role of oral prednisolone in wheezing induced by viral infection [4].

They have randomly treated 687 preschool children with an attack of wheezing associated with viral infection with either prednisolone or placebo for 5-days.

The results of this study definitely showed that oral corticosteroids cannot be proposed as an effective treatment in children with viral induced wheezing and, therefore, it no longer allows the indiscriminate use of such a common treatment in this situation, at least in mild-to-moderate conditions.

As clearly pointed in the accompanying editorial by Bush [5], the results of this study are to be considered in the frame of the characteristics of the study population, i.e. preschool children who didn't present with the classic atopic asthma phenotype, which, on the other hand, is positively recognized to be responsive to corticosteroid treatment.

Therefore, even if it has to be taken into consideration that also severe episodic viral wheezing may need a treatment with oral steroid, the evidence from the study by Panickar and co-workers no longer justify the use of oral steroids in non-atopic children for whom a severe prognosis is not anticipated [5]

Inhaled corticosteroid courses are proposed even more frequently than oral ones in children presenting with wheeze.

Ducharme et al. have demonstrated that high dose inhaled fluticasone (750 mcg/bid) can be somewhat effective in preventing the use of oral corticosteroids in preschool children with moderate-to-severe virus -induced wheezing [6]. The apparent paradox of a better response to the administration of an inhaled corticosteroid in a population with clinical characteristics very similar to those of the group receiving oral prednisone in the Panickar's study could be speculatively explained by a vasoconstrictive effect which is potentially resulting in an anti-edema effect at the site of the bronchial mucosa [5], rather than due to an anti-inflammatory effect.

Nevertheless, the Authors observed a potential risk of side effects on growth in children treated with a such high dose of fluticasone and clearly warned about the use of this therapeutic strategy in children.

On the basis of the hypothesis of an anti-oedema effect of high doses of inhaled corticosteroid, other drugs with a vasoconstrictive effect could be even more effective in controlling viral wheezing, with potentially lower risk of side effects, thus warranting further studies with the aim to address this specific question.

If the issue of using corticosteroid in acute episodes of wheezing in preschool children deserved the publication of the two above reported studies, a meta-analysis aiming to evaluate the efficacy of prolonged treatment with inhaled corticosteroids in preschool children with recurrent episodes of wheezing or asthma was recently published in the Pediatrics [7].

The Authors considered 29 randomized trials, including 3.592 children who were receiving for at least four weeks of inhaled corticosteroids for their frequent wheezing episodes. They concluded that ICS are useful in such paediatric population in terms of reduction of exacerbations, symptoms and lung function improvement.

Nevertheless, though the conclusions of this analysis are presented for the pooled group of wheezing and asthmatic children, they are mostly driven by studies including patients with pronounced asthmatic features as previously described by Castro-Rodriguez et al. [2], for whom the effectiveness of ICS treatment is widely accepted.

In particular, the study by Guilbert et al [8], which weighted 22,8% for the RR of wheeze/asthma exacerbation (WAE), was designed to select and treat children with a positive asthma predictive index.

Also in the study by Baker et al. [9], which weighted for 15.1% in the meta-analysis by Castro Rodriguez and Rodrigo [7], were considered only children with a specific diagnosis of persistent asthma.

In the study by Roorda et al [10], accounting for 13.7% in the meta-analysis, it is clearly indicated that a significant response to ICS treatment was observed in the children with frequent symptoms, a family history of asthma, or both, but not in those without a family history. Similar consideration can be extended to a number of further studies with lighter weight in the analysis.

Therefore, the conclusion that ICSs are useful in infants and preschool children with wheeze/asthma can represent a misleading message to the reader regarding the potential usefulness of a maintenance treatment with ICSs in the group of transient wheezers.

To the best of our knowledge, for this group, at present, there is no indication that a maintenance treatment with ICCs can be effective in reducing the number or the severity of wheezing [11].

## Conclusions

The main clinical dilemma for paediatricians dealing with preschool children with wheezing is represented by the difficulty to predict the future development of the disease in the individual patients. At the present we know that the response to systemic and inhaled steroids in this age group is poor, due to the prevalence of viral wheeze in these children[12].

Therefore, at present, the therapeutic options potentially available for preschool children with episodic non-atopic wheeze are β2-agonists to control the re-exacerbations, along with intermittent or prophylactic leukotriene receptors antagonists, whereas high dose inhaled corticosteroids are not recommended and oral corticosteroids should be considered only in severe children in hospital setting [5].

We agree with Saglani and Bush that asthma in preschool children represents the next challenge for pediatricians and that there is a desperate need for both a more efficient phenotypic process and new therapeutic options for these children [13].
